# Mechanism of miR-4465 targeting PTEN-mediated autophagy of astrocytes in epilepsy

**DOI:** 10.5937/jomb0-52626

**Published:** 2025-09-05

**Authors:** Jinhua Zhao, Jihong Tang, Xiaoyan Shi

**Affiliations:** 1 Children's Hospital Affiliated to Soochow University, Department of Neurology, Suzhou, Jiangsu, China

**Keywords:** astrocytes, miR-4465, PTEN, autophagy, astrociti, miR-4465, PTEN, autofagija

## Abstract

**Background:**

To investigate the mechanism of miR-4465 targeting PTEN-mediated autophagy of astrocytes in epilepsy.

**Methods:**

Serum samples were collected from epileptic children and healthy children. Extract foreign bodies from serum samples and determine their quality. The exosomes were sequenced, and the abnormal expression of miRNA in patients' serum exosomes was analysed, and the expression of miR-4465 was verified by quantitative PCR. Bioinformatics predicts the size of miR-4465 and makes GO and KEGG analyses. HEK293 cells were cultured, and the relationship between miR-4465 and its target was detected using the double luciferase reporting method. Astrocytes were cultured, and quantitative PCR and WB were used to detect the expression of miR-4465 and PTEN after overexpression. In addition, CCK-8 and WB were used to detect the growth of miR-4465 and the changes of autophagy-related proteins ATG5 and Beclin1, respectively.

**Results:**

miR-4465 was markedly increased in exosomes. The bioinformatic analysis found the differentially expressed target genes of miR-4465 were mainly enriched in molecular binding, molecular function regulation, and other molecular functions and participated in cell adhesion, cell-extracellular matrix receptor interaction, and the Rap1 signalling pathway. PTEN has been predicted as a target gene of miR-4465; meanwhile, the results of the dual-luciferase reporter assay confirmed the interaction between miR-4465 and PTEN. Quantitative PCR, as well as WB results, suggested the level of PTEN was decreased in serum exosomes of patients with epilepsy, while increased miR-4465 expression inhibited expressions of PTEN. CCK-8, as well as WB results, suggested miR-4465 could suppress the growth of astrocytes and promote ATG5 as well as Beclin1 expression; finally, up-regulation of PTEN partially alleviated effects of miR-4465 on astrocytes growth as well as autophagy.

**Conclusions:**

In children with epilepsy, miR-4465 can target and regulate PTEN to promote autophagy in astrocytes.

## Abstract (sr)

**Uvod:**

Cilj je bio da se ispita mehanizam delovanja miR-4465 na PTEN-posredovanu autofagiju astrocita kod epilepsije.

**Metode:**

Prikupljeni su uzorci seruma od dece obolele od epilepsije i od zdrave dece. Iz uzoraka seruma ekstrahovana su strana tela i određen je njihov kvalitet. Egzomi su sekvencirani, analizirana je abnormalna ekspresija miRNA u egzosomima seruma pacijenata, a ekspresija miR-4465 je verifikovana kvantitativnim PCR-om. Bioinformatičkom analizom je predviđena veličina miR-4465, uz sprovođenje GO i KEGG analize. Ćelije HEK293 su kultivisane, a odnos između miR-4465 i njegove ciljne molekule detektovan je metodom dvostrukog luciferaznog reporter testa. Astrociti su kultivisani, a kvantitativnim PCR-om i WB metodom detektovana je ekspresija miR-4465 i PTEN nakon njihovog prekomernog izražavanja. Pored toga, CCK-8 i WB metodom praćen je rast miR-4465 i promene proteina ATG5 i Beclin1 u vezi sa autofagijom.

**Rezultati:**

Ekspresija miR-4465 je bila značajno povećana u egzosomima. Bioinformatičkom analizom otkriveno je da su diferencijalno izraženi ciljni geni miR-4465 uglavnom obogaćeni u funkcijama molekularnog vezivanja, molekularnih funkcija regilacije i drugih molekularnih funkcija, te da učestvuju u ćelijskoj adheziji, interakciji ćelija sa receptorima vanćelijskog matriksa i Rap1 signalnog puta. PTEN je predviđen kao ciljni gen miR-4465, a rezultati dvostrukog luciferaznog reporter testa potvrdili su interakciju između miR-4465 i PTEN-a. Kvantitativni PCR i WB rezultati sugerišu da je nivo PTEN-a smanjen u egzosomima seruma pacijenata sa epilepsijom, dok povećana ekspresija miR-4465 inhibira ekspresiju PTEN-a. CCK-8 i WB rezultati ukazuju da miR-4465 može suprimirati rast astrocita i podstaći ekspresiju ATG5 i Beclin1. Na kraju, regulacija PTEN-a naviše delimično ublažava efekte miR-4465 na rast astrocita i autofagiju.

**Zaključak:**

Kod dece sa epilepsijom, miR-4465 može ciljati i regulisati PTEN kako bi podstakao autofagiju u astrocitima.

## Introduction

Epilepsy has been identified as a commonly observed neuron disease in children [1]. The incidence and prevalence of childhood epilepsy in China exhibit specific characteristics. According to epidemiological data, there are approximately 6 million epilepsy children in China, meaning that 7 out of every 1,000 Chinese children have epilepsy [2] [3]. This condition is caused by excessive abnormal discharges in the cerebral cortex due to stimulation, and the causes of epilepsy were either primary or secondary. Primary epilepsy is often associated with genetics, whereas secondary epilepsy is triggered by other diseases or brain injuries [4].

Studies have shown that dysfunction of astrocytes is one of the key factors leading to neuronal overexcitement and epileptic seizures. The metabolism and function of astrocytes become vigorous during epileptic seizures [5]. Results of some recent works suggested the functions of astrocytes may be regulated by microRNAs (miRNAs) [6]. miRNAs belong to the non-coding RNA family, and miRNA dysfunction may lead to neurological diseases [7], i.e. temporal lobe epilepsy [8]. In the pathogenesis of epilepsy, highly synchronised abnormal discharges of neurons can cause brain injury, and autophagy may participate in this process [9] [10].

On the other hand, miRNAs may serve as important regulators in the autophagy process [11], and miR-4465 can inhibit cellular autophagy [12]. PTEN (phosphatase and tensin homolog) has been reported to participate in various biological events, especially in regulating cell proliferation, migration, and survival. PTEN is widely expressed in the central neuron system. A previous report suggested that miR-4465 directly act on PTEN and inhibits AKT-mTOR-induced autophagy [12]. Nevertheless, mechanisms of miRNAs in regulating PTEN-related autophagy in childhood epilepsy are still unclear. In the current work, we will investigate the roles of miR-4465 in regulating PTEN-mediated autophagy in epilepsy and explore its molecular mechanism.

## Materials and methods

### Patients and cells

Cells: HEK293 cells and astrocytes were purchased from the Chinese Academy of Sciences cell bank. Cells were cultured using F12 with 1×105 U/L penicillin, 100 mg/mL streptomycin, and 10% FBS. They were incubated at 37°C and 5% CO2. Astrocytes were treated after 3 days of culture.

Sample size calculation: We calculate the required sample size according to the formula: n=, so that the confidence level is 95%, the z value is 1.96, and the allowable error limit is 5% (E=0.05). In China, the incidence of children with epilepsy is about 0.7% [2]. The calculated sample size is 33 cases. Sixty children with epilepsy were finally included in this study, which met the sample size requirements of this part of the study on influencing factors.

Sample information: All serum samples were collected from 60 children with simple epilepsy admitted to the Children’s Hospital affiliated with Soochow University as the epilepsy group. The clinical case information of all patients is complete, including 32 males and 28 females, age 6|15 (8.78±2.06) years old; height 113|159 (138.45±14.60) cm. The onset age is 1|5 (2.27±0.48) years old; 21 cases had a disease course of less than 3 years, 39 cases had a disease course of ≥ 3 years, 22 cases had an onset time of less than 5 minutes, and 39 cases had a disease course of ≥ 5 minutes; 36 cases had a frequency of less than 3 attacks per year, and 21 cases had a frequency of more than 3 attacks per year. During the same period, 60 healthy children were selected from the physical examination centre of Suzhou University Children’s Hospital as the control group, including 27 males and 38 females, age 6|16 years old (8.94+2.25) years old; height: 115|163 (136.72±14.73) cm. There was no statistically significant difference in gender and age between the two groups (P>0.05), and all guardians signed informed consent forms.

### Exosome extraction

The serum samples have been centrifuged (3,000×g, 15 minutes), and the supernatant has been transferred to a clean EP tube on ice, taking care not to disturb the clump-like precipitate in the supernatant. Then, 0.2 times the volume of the exosome extraction reagent (Umibio UR52121) was added to the samples. The sample/exosome extraction reagent mixture was gently shaken or vortexed until the homogeneous solution was formed, and then incubated (4°C, 30 minutes) and centrifuged (10,000×g, 10 minutes) at room temperature. Then, the supernatant was discarded, and the precipitates were resuspended in an appropriate amount of PBS buffer or a similar buffer. The extracted total exosomes were stored at -20°C.

Observing the extracellular vesicle sample through transmission electron microscopy, 10 μL of the sample was dropped onto a copper mesh and precipitated for 60 seconds. The float was removed by filter paper, and then 10 μL of uranyl acetate was dropped onto the copper mesh and precipitated for 60 seconds. After air-drying for a few minutes, the sample was observed and imaged under a 100 kV electron microscope.

### Exosome RNA extraction

The exosomes have been centrifuged (3,000×g, 15 minutes), and the supernatants have been aspirated. Then 1 mL of TRIzol reagent was added, the lysis solution was transferred to the 1.5 mL RNase-free EP tube, and 200 μL of chloroform was added for 10 minutes. Next, the samples were centrifuged, and a new EP tube was prepared with 500 μL of isopropanol pre-chilled. Fifteen minutes after the centrifugation step, the aqueous phase of the samples was transferred and precipitated using alcohol for 10 minutes. Then, the samples were centrifuged (13,000 rpm, 10 minutes), and RNA precipitates were washed using 75% ethanol and then centrifuged (12,000 rpm, 5 minutes). Next, RNA precipitates have been air-dried or vacuum-dried and dissolved using DEPC-treated deionised water. Finally, the concentration and purity of the samples were determined by spectrophotometric analysis.

### miRNA sequencing

Firstly, the total RNA sample was analysed for concentration and integrity using the Agilent 2100 Bioanalyzer. Small RNAs were isolated from qualified total RNAs and enriched, then linked to single-strand DNA for hybridisation amplification. The by-products were removed by polyacrylamide gel electrophoresis (PAGE), and the target bands after PCR were recovered to construct the library. Quantification of the constructed library was performed using the Agilent 2100 Bioanalyzer. The double-stranded PCR products were denatured into single strands and then cyclised with primers. DNA nanospheres were formed by phi29 enzyme action. The DNA molecular anchor and fluorescent probe were polymerised with the DNA nanospheres using a combined probe anchoring polymerisation technique, and miRNA sequencing was completed using the BGISEQ-500 sequencing platform.

Filter and standardise the raw data after sequencing, compare the processed data with reference genomes and other small RNA databases using AASRA alignment software and standardise the expression levels of small RNAs using the Transcript Per Million (TPM) algorithm. The default screening criteria for differentially expressed genes are absolute difference fold change | log2 (fold change) | ≥ 2 and Q value <0.01. Perform hierarchical clustering analysis on the screening results.

Perform target gene prediction on the selected differentially expressed miRNAs, and ultimately select target genes with an intersection of ≥ 3 in the database and regulated by miRNAs of ≥ 2 as the prediction results. Based on the prediction results, perform gene ontology (GO) and Kyoto Encyclopedia of Genes and Genomes (KEGG) enrichment analysis to further understand the signalling pathways and biological functions affected by differentially expressed genes.

### Quantitative PCR

The total RNAs were isolated using Trizol reagent (Invitrogen, USA) and quantified using a Qubit fluorometer. Then, the total RNAs were reverse-transcripted by a High-Capacity cDNA Reverse Transcription Kit (Vazyme MR101-01). The PCR reaction system was prepared by adding the reverse-transcribed cDNA and primers to the PCR reaction tubes. PCR was performed using the ABI-7300 fluorescent quantitative PCR instrument under 95°C 10 s, 60°C 20 s, and 72°C 15 s, repeated for 40 cycles. The quantitative detection reagent kit (Vazyme MQ101-01) was used. The sequences of the primers have been presented in Table 1. U6 has been applied as the reference gene for miR-4465, while GAPDH has been applied as the reference gene for PTEN. The relative miR-4465, as well as PTEN expressions, have been evaluated using the 2-^ΔΔ^CT method and expressed as mean ± standard deviation.

**Table 1:** Primer sequences.

Gene	Sequences
miR-4465	F:CGCGCTCAAGTAGTCTGACC<br>R:AGTGCAGGGTCCGAGGTATT
U6	F:CTCGCTTCGGCAGCACA<br>R:AACGCTTCACGAATTTGCGT
PTEN	F: CCGAAAGGTTTTGCTACCATTCT<br>R: AAAATTATTTCCTTTCTGAGCATTCC
GAPDH	F: GTCAACGGATTTGGTCTGTATT<br>R: AGTCTTCTGGGTGGCAGTGAT

### Dual-luciferase reporter assay

A reporter plasmid containing PTEN 3’UTR region with the miR-4465 binding sequence and a mutant plasmid were constructed. HEK293 cells have been seeded into 12-well cell culture plates, and wildtype, as well as mutant plasmids, have been transfected with either miR-4465 mimics or negative control. Then, 24 hours after transfection, cells were collected, and the activities of firefly and Renilla luciferase of each group were determined according to the instructions of the kit. Results have been statistically analysed using firefly/Renilla luciferase activities.

### Cell transfection

The transfection mixture has been prepared in sterile EP tubes. For solution A, 200 nmol/L miR-4465 mimics were dissolved in Opti-MEM (200 μL). For solution B, 5 μL Lipo2000 was dissolved Opti-MEM (200 μL). Solutions A and B were gently mixed separately and were let to stand for 5 minutes, then solution B was added to solution A and was gently mixed for 20 minutes. Then, a transfection reagent was added, and 6 hours later, it was replaced with a complete culture medium and was continued to be cultured for 24–|48 hours. The efficacy of transfection was detected by the immunofluorescent method.

### Western blot

RIPA lysis buffer (Solarbio) was used to extract proteins from cells. SDS-PAGE electrophoresis: The concentration of the proteins was examined using a BCA kit, and then electrophoresis was conducted on a 12% SDS-PAGE gel, loading 40 μg of protein per well. After electrophoresis at 80 V for 120 minutes, the proteins were transferred onto a PVDF membrane, treated with 300 mA for 90 minutes, and then blocked with 5% non-fat milk for 60 minutes at room temperature. Then, the target protein bands were cut according to the marker position and placed in incubation boxes. Rabbit primary antibodies against CD63 (1:1000, CST, 52090), CD81 (1:1000, CST, 56039), PTEN (1:1000, CST, 9559), Beclin1 (1:1000, CST, 3945), ATG5 (1:1000, CST, 12994), and mouse primary antibody against GAPDH (1:1000, CST, 97166) were added. On day 2, the membranes were washed, and horseradish peroxidase- conjugated antibodies (1:10000, ComWin Biotech Co., Ltd., China) were added. It was then incubated on a shaker at 70–80 rpm. Then, the signal of the secondary antibody bound to the primary antibody was detected using ECL detection reagents and a chemiluminescence detection system. The density ratio of the target band to GAPDH was analysed using ImageJ software.

### CCK-8 cell viability assay

Astrocytes were transfected and seeded on 96-well plates with 2 × 10^3^ cells per well for 6 hours. Cell attachment was observed, and culturing was continued for 0, 24, 48, or 72 hours. CCK-8 solution (10 μL) was placed in each well for 4 hours, and a microplate reader measured absorbance (450 nm).

### Statistics

Statistics have been conducted by SPSS 22.0. All data have been expressed as mean ± standard deviation. For two-group comparisons, a t-test was applied, and for more than group comparisons, a one-way analysis of variance (ANOVA) was used. A p-value of less than 0.05 has been considered statistically significant.

## Results

### Quality of extracellular vesicle samples

**Figure 1:**
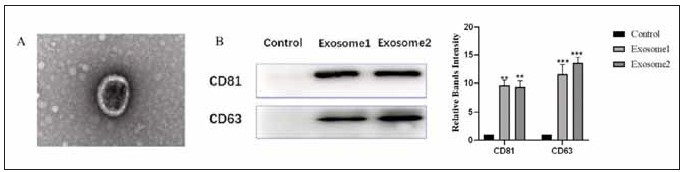
Identification of extracellular vesicle quality.<br>A: Observation of extracellular vesicle structure by transmission electron microscopy. B: Western blot analysis of CD63 and CD81 expression in extracellular vesicles, compared with the control group, * P<0.05, ** P<0.01. ***P<0.001.

To determine whether the extracted extracellular vesicle samples met the quality requirements, we first observed their structure and shape. The extracellular vesicles have a normal horseshoe-shaped morphology (Figure 1A). Moreover, D63 and CD81 were detected in the extracted extracellular vesicles, indicating that they were of suitable quality (Figure 1B).

### miR-4465 is highly expressed in blood extracellular vesicles of pediatric epilepsy patients

**Figure 2:**
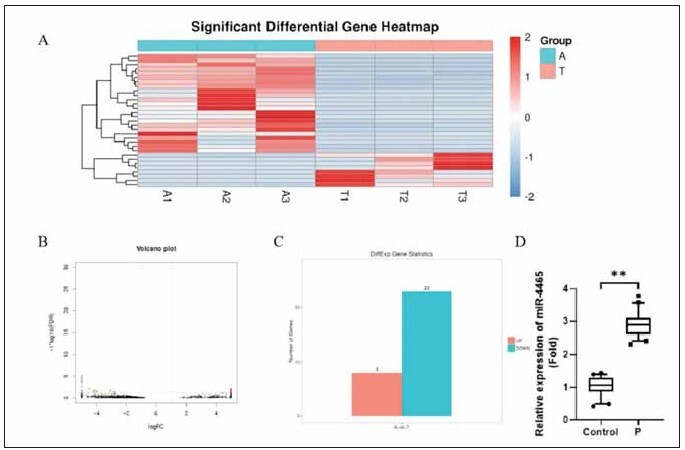
Expression of miR-4465 in extracellular vesicles from pediatric epilepsy patients.<br>A: heat map; B: volcano map; C: statistical chart of differences and analysis of differences in exosomes miRNA-seq. D: Quantitative PCR validation of miR-4465 expression in extracellular vesicles. ** P<0.01.

To screen abnormally expressed miRNAs in patient serum extracellular vesicles, we performed miRNA sequencing on the extracted extracellular vesicles. Sequencing analysis revealed that relatively few miRNAs were abnormally expressed in patient serum extracellular vesicles. Compared to the control group, eight miRNAs were upregulated, and 23 miRNAs were down-regulated (Figure 2A-C), among which the small RNA miR-4465 was upregulated. We then validated miR-4465 expression by quantitative PCR, and we found the levels of miR-4465 significantly increased in patient serum extracellular vesicles (Figure 2D, p<0.05).

### miR-4465 inhibits astrocyte proliferation and promotes autophagy

**Figure 3:**
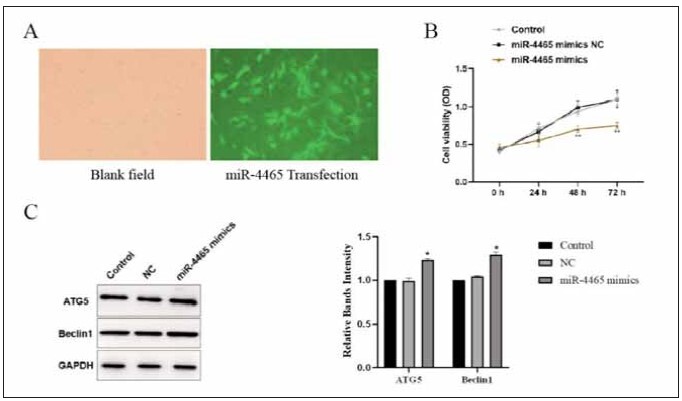
Effects of miR-4465 on astrocyte proliferation and autophagy.<br>A: Transfection efficiency. B: CCK-8 assay to detect the effect of miR-4465 overexpression on cell viability. C: Western blot analysis of the effects of miR-4465 overexpression on the expression of autophagy-related proteins, compared with the control group, * *P*<0.05, ** *P*<0.01, ***P<0.001

To investigate the effects of miR-4465 on the growth and autophagy of astrocytes, we transfected astrocytes. We used a CCK-8 assay kit to detect changes in cell viability among the groups, and the transfection efficiency is shown in Figure 3A. As Figure 3 shows, miR-4465 over-expression markedly decreased cell viability (Figure 3B, *p*<0.05), indicating that over-expression of miR-4465 inhibits cell proliferation. We then performed western blot analysis to examine autophagy-related protein ATG5 as well as Beclin-1 expression. We found the levels of ATG5 and Beclin1 were significantly increased in cells overexpressing miR-4465 (Figure 3C, *p*<0.05).

### PTEN is a target of miR-4465

To study the function and evaluate the possible targets of miR-4465, we first performed a bioinformatic analysis. We then conducted GO and KEGG analyses on these target genes. The GO analysis results suggested the differently expressed genes (DEGs) were mainly enriched in molecular binding, molecular function regulation, and biological processes such as growth and development (Figure 3A). KEGG analysis indicated the DEGs might participate in pathways such as cell adhesion, extracellular matrix-receptor interaction, NF-kappa B signalling, B-cell receptor signalling, PI3K-Akt signalling, as well as Rap1 signalling (Figure 3B). Furthermore, TargetScan predicted that PTEN might be a target gene of miR-4465 (Figure 3C). Finally, a dual-luciferase reporter assay was conducted to evaluate the correlation between miR-4465 and PTEN. We found the activity of the luciferase was markedly deduced in miR-4465 mimics + wild-type PTEN reporter plasmid transfected cells, and the activity of the luciferase did not change in cells that were treated by miR-4465 mimics as well as mutant PTEN reporter plasmid, suggesting the targeting relationship between miR-4465 and PTEN (Figure 3D, *p*<0.05).

### miR-4465 over-expression inhibits PTEN mRNA and protein expression

**Figure 4:**
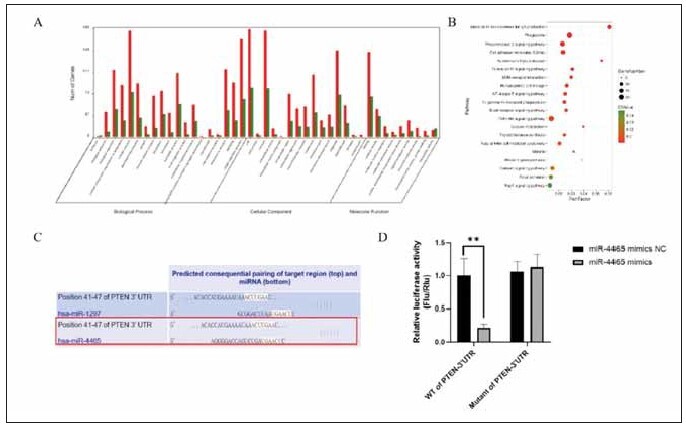
Prediction and validation of the miR-4465 target gene.<br>A–B: GO analysis to study gene function. B: KEGG analysis to study metabolic pathways. C: TargetScan analysis predicting PTEN as a potential target gene of miR-4465. D: Dual-luciferase reporter assay validating the targeting relationship between miR-4465 and PTEN. ** *P*<0.01.

**Figure 5:**
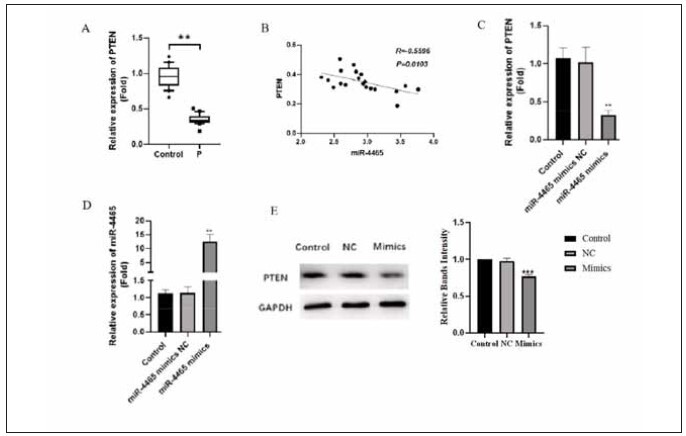
Regulation of PTEN expression by miR-446.<br>A: Quantitative PCR detection of PTEN expression in extracellular vesicles. B: Correlation of PTEN and miR-4465 expression. C-D: Quantitative PCR detection of miR-4465 and PTEN expression in cells after miR-4465 transfection. E: Western blot analysis of PTEN expression in cells after miR-4465 mimic transfection, compared with the control group, * *P*<0.05, ** *P*<0.01, ***P<0.001.

To investigate the relationship between miR-4465 and PTEN expression, we first performed quantitative PCR to detect PTEN expression in extracellular vesicles. We found the expression of PTEN was markedly reduced (Figure 4A, *p*<0.01), and the PTEN as well as miR-4465 expression was negatively correlated (Figure 4B, *p*<0.05). We then performed quantitative PCR to detect miR-4465 and PTEN expression in cells after miR-4465 transfection. The results showed that levels of miR-4465 were markedly elevated (Figure 4C, *p*<0.01), while levels of PTEN significantly reduced (Figure 4D, *p*<0.01) in cells transfected with miR-4465 mimics. Finally, WB results suggested the levels of PTEN were markedly reduced in cells transfected with miR-4465 mimics (Figure 4E, *p*<0.001). Figure 5

### miR-4465/PTEN regulates astrocyte cell growth as well as autophagy

**Figure 6:**
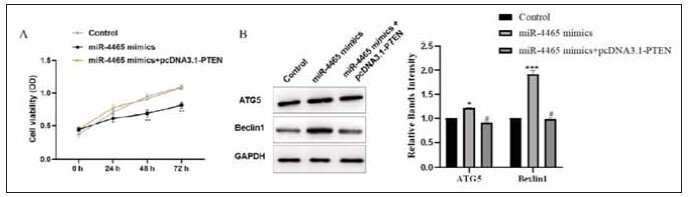
Effects of miR-4465/PTEN on cell proliferation and autophagy.<br>A: CCK-8 assay to detect cell viability. B: Western blot analysis of the expression of autophagy-related proteins,* *P*<0.05, *** *P*<0.001 in comparison to the control group, #P<0.05 in comparison to the miR-4465 mimics group.

To evaluate the effects of PTEN on growth as well as autophagy, we transfected astrocytes and used the CCK-8 assay kit to detect changes in cell viability among the groups. As Figure 6 shows, miR-4465 over-expression markedly suppressed cell viability, and the inhibition effect of miR-4465 over-expression was significantly alleviated by upregulating PTEN expression (Figure 6A, *p*<0.05), suggesting that PTEN promotes cell proliferation. We then performed WB analysis to evaluate ATG5 and Beclin-1 expressions. We found that ATG5 and Beclin1 were elevated in cells that are over-expressing miR-4465, while the effect was partially alleviated by upregulating PTEN expression (Figure 6B, *p*<0.001), suggesting that PTEN inhibits autophagy in cells.

## Discussion

Epilepsy affects over 70 million people globally [4], and it is a leading cause of disability among children [13]. The pathogenesis of epilepsy involves multiple factors, such as brain structural abnormalities, genetic predisposition, brain injury, and infections. Moreover, external stimuli like light, sound, and emotional changes can also trigger epileptic seizures [14]. Although the clinical manifestations of childhood epilepsy vary, common symptoms include recurrent muscle spasms, impaired consciousness, sensory abnormalities, and transient emotional disturbances [15]. Due to the patient population being children, the available treatment options are relatively limited, posing various challenges in the treatment process.

Extracellular vesicles possess potent intercellular communication functions and play crucial roles. Specific proteins or miRNAs in extracellular vesicles are highly associated with epilepsy [16]. MicroRNAs can bind to the complementary sites of the mRNAs, decreasing their stability, downregulating translation, and regulating gene expressions [17]. In recent years, results of previous studies revealed the differential expressions of various miRNAs in the serum of epilepsy patients [18]. miR-155 is upregulated in children with epilepsy and is associated with the severity of epilepsy [19].

Additionally, miR-194-5p [20] and miR-148a-3p [21] are significantly dysregulated in the plasma of children with temporal lobe epilepsy. In the present study, we extracted extracellular vesicles from the serum of children with epilepsy. We confirmed a significant increase in miR-4465 expression in these extracellular vesicles through miRNA sequencing analysis and PCR. Previous studies have revealed the important role of miR-4465 in many cancers, such as cervical cancer and ovarian cancer [22] [23]. In these studies, miR-4465 has been proven to inhibit the proliferation, migration and invasion of tumour cells. In the current study, the function of miR-4465 has been further extended to the nervous system, especially astrocytes.

Autophagy is a highly conserved degradation process that delivers dysfunctional proteins, lipids, and organelles to lysosomes for degradation [12]. In recent years, more and more studies have shown that autophagy plays an important role in nervous system diseases, including epilepsy [24] [25]. Astrocytes are located between the cell bodies and processes of neurons [26], and they play vital roles in the central nervous system. Reports indicate that astrocytes are crucial in the development of epilepsy, and alterations in their ability to uptake glutamate or -aminobutyric acid can lead to epileptic seizures [27]. Therefore, astrocytes are commonly used in the study of neurological diseases. In current work, astrocytes have been cultured, and the results suggested miR-4465 can inhibit cell proliferation and promote autophagy, suggesting that miR-4465 may play vital roles in childhood epilepsy.

Some studies suggested that miR-4465 can promote autophagy by targeting the PTEN gene [12]. PTEN servers are tumour suppressor genes and may participate in the process of maintaining cell proliferation and differentiation [28]. PTEN can modulate neuronal excitability, synaptic transmission, and synaptic plasticity by regulating intracellular signalling pathways, and abnormalities in these processes may lead to neuronal hyperexcitability and trigger epileptic seizures [29]. Additionally, PTEN has also been found to be involved in the development and function of the nervous system, including regulating neuron survival, synaptic plasticity and learning and memory [26]. This study links PTEN with the autophagy of astrocytes, which provides a new perspective for understanding the new functions of PTEN in the nervous system. To further evaluate the mechanism by which miR-4465 mediates autophagy in astrocytes, we also identified and validated the correlation between miR-4465 and PTEN. The result was consistent with Roquid et al.’s [30] finding. Similarly, we found that PTEN expression was down-regulated in the extracellular vesicles of children with epilepsy and negatively correlated with miR-4465 expression. To investigate the effects of miR-4465 mimics on PTEN, we performed cell-based experiments. We found that miR-4465 over-expression could inhibit PTEN expression while upregulating PTEN expression partially counteracted the effects of miR-4465 mimics on growth as well as autophagy of the cells. These results indicate that miR-4465 can target PTEN to regulate autophagy in astrocytes.

To sum up, the current research reveals the new mechanism of miR-4465 targeting PTEN-mediated astrocyte autophagy, which provides new potential targets and ideas for epilepsy treatment. However, further experiments and clinical studies are needed to validate their precise mechanisms of action and clinical significance.

## Dodatak

### Conflict of interest statement

All the authors declare that they have no conflict of interest in this work.

## References

[1] Fine A, Wirrell E C (2020). Seizures in Children. Pediatr Rev.

[2] Song P, Liu Y, Yu X, Wu J, Poon A N, Demaio A, Wang W, Rudan I, Chan K Y (2017). Prevalence of epilepsy in China between 1990 and 2015: A systematic review and meta-analysis. J Glob Health.

[3] Guerrini R (2006). Epilepsy in children. Lancet.

[4] Thijs R D, Surges R, O'Brien T J, Sander J W (2019). Epilepsy in adults. Lancet.

[5] Abduljabbar R, Tammimi D E, Yousef A - M, Mukred S R, Zawiah M (2023). SCN1A polymorphisms influence the antiepileptic drugs responsiveness in Jordanian epileptic patients. J Med Biochem.

[6] Gorina Y V, Salmina A B, Erofeev A I, Gerasimov E I, Bolshakova A V, Balaban P M, Bezprozvanny I B, Vlasova O L (2022). Astrocyte Activation Markers. Biochemistry (Mosc).

[7] Cao D D, Li L, Chan W Y (2016). MicroRNAs: Key Regulators in the Central Nervous System and Their Implication in Neurological Diseases. Int J Mol Sci.

[8] Gorter J A, Iyer A, White I, Colzi A, van Vliet E A, Sisodiya S, Aronica E (2014). Hippocampal subregion-specific microRNA expression during epileptogenesis in experimental temporal lobe epilepsy. Neurobiol Dis.

[9] Giorgi F S, Biagioni F, Lenzi P, Frati A, Fornai F (2015). The role of autophagy in epileptogenesis and in epilepsy-induced neuronal alterations. J Neural Transm (Vienna).

[10] Zhao Y, Zhao W, Han Y (2023). Inhibition of mTORC2 improves brain injury in epileptic rats by promoting chaperone-mediated autophagy. Epilepsy Res.

[11] Ghafouri-Fard S, Shoorei H, Mohaqiq M, Majidpoor J, Moosavi M A, Taheri M (2022). Exploring the role of non-coding RNAs in autophagy. Autophagy.

[12] Tao Z, Feng C, Mao C, Ren J, Tai Y, Guo H, Pu M, Zhou Y, Wang G, Wang M (2019). MiR-4465 directly targets PTEN to inhibit AKT/mTOR pathway-mediated autophagy. Cell Stress Chaperones.

[13] Huang W S, Zhu L (2018). MiR-134 expression and changes in inflammatory cytokines of rats with epileptic seizures. Eur Rev Med Pharmacol Sci.

[14] Chen Z, Brodie M J, Liew D, Kwan P (2018). Treatment Outcomes in Patients With Newly Diagnosed Epilepsy Treated With Established and New Antiepileptic Drugs: A 30-Year Longitudinal Cohort Study. JAMA Neurol.

[15] Fisher R S, Acevedo C, Arzimanoglou A, Bogacz A, Cross J H, Elger C E, Engel J Jr, Forsgren L, French J A, Glynn M, et al (2014). ILAE Official Report: A practical clinical definition of epilepsy. Epilepsia.

[16] Raoof R, Bauer S, El Naggar H, Connolly N M C, Brennan G P, Brindley E, Hill T, McArdle H, Spain E, Forster R J, et al (2018). Dual-center, dual-platform microRNA profiling identifies potential plasma biomarkers of adult temporal lobe epilepsy. EBioMedicine.

[17] Lu T X, Rothenberg M E (2018). MicroRNA. J Allergy Clin Immunol.

[18] Ma Y (2018). The Challenge of microRNA as a Biomarker of Epilepsy. Curr Neuropharmacol.

[19] Liu Y, Yu G, Ding Y, Zhang Y X (2022). Expression of miR-155 in Serum Exosomes in Children with Epilepsy and Its Diagnostic Value. Dis Markers.

[20] Niu X, Zhu H L, Liu Q, Yan J, Li M L (2021). MiR-194-5p serves as a potential biomarker and regulates the proliferation and apoptosis of hippocampus neuron in children with temporal lobe epilepsy. J Chin Med Assoc.

[21] Yu Y, Du L, Zhang J (2021). Febrile Seizure-Related miR-148a-3p Exerts Neuroprotection by Promoting the Proliferation of Hippocampal Neurons in Children with Temporal Lobe Epilepsy. Dev Neurosci.

[22] Tang X, Wen X, Li Z, Wen D, Lin L, Liu J, Li M (2021). Hsa_circ_0102171 aggravates the progression of cervical cancer through targeting miR-4465/CREBRF axis. J Cell Physiol.

[23] Liu J, Zeng Y, Zheng L (2023). Up-regulation of oxidised low-density lipoprotein receptor 1 correlates with decreased miR-106b-5p, miR-93-5p, miR-3129-5p, miR-199b-3p, and miR-4465, higher recurrence rate, and poor prognosis in ovarian cancer. Histol Histopathol.

[24] Fassio A, Falace A, Esposito A, Aprile D, Guerrini R, Benfenati F (2020). Emerging Role of the Autophagy/Lysosomal Degradative Pathway in Neurodevelopmental Disorders With Epilepsy. Front Cell Neurosci.

[25] Chen C, Zhu T, Gong L, Hu Z, Wei H, Fan J, Lin D, Wang X, Xu J, Dong X, et al (2023). Trpm2 deficiency in microglia attenuates neuroinflammation during epileptogenesis by upregulating autophagy via the AMPK/mTOR pathway. Neurobiol Dis.

[26] Verkhratsky A, Nedergaard M (2018). Physiology of Astroglia. Physiol Rev.

[27] Vezzani A, Ravizza T, Bedner P, Aronica E, Steinhäuser C, Boison D (2022). Astrocytes in the initiation and progression of epilepsy. Nat Rev Neurol.

[28] Álvarez-Garcia V, Tawil Y, Wise H M, Leslie N R (2019). Mechanisms of PTEN loss in cancer: It's all about diversity. Semin Cancer Biol.

[29] Dhamija R, Hoxworth J M (2020). Imaging of PTEN-related abnormalities in the central nervous system. Clin Imaging.

[30] Roquid K A R, Alcantara K M M, Garcia R L (2020). Identification and validation of mRNA 3'untranslated regions of DNMT3B and TET3 as novel competing endogenous RNAs of the tumor suppressor PTEN. Int J Oncol.

